# Integrated Hydrological and Geophysical Characterisation of Surface and Subsurface Water Contamination at Abandoned Metal Mines

**DOI:** 10.1007/s11270-018-3880-4

**Published:** 2018-07-17

**Authors:** Emily Hudson, Bernd Kulessa, Paul Edwards, Tom Williams, Rory Walsh

**Affiliations:** 1 College of Science, Swansea University, Singleton Park, Swansea, Wales SA2 8PP UK; 2 Natural Resources Wales, Maes Newydd, Llandarcy, Neath Port Talbot SA10 6JQ UK

**Keywords:** Flow gauging, Geophysics, Heavy metals, Hydrological contamination, Water quality

## Abstract

The mining and processing of metal ores in the UK has left a legacy of environmental degradation, and abandoned metal mines still pose a significant threat to terrestrial and fluvial environments. Flow gauging, water quality and geophysics were combined in an integrated assessment of surface and subsurface hydrological contamination at Esgair Mwyn, an abandoned mine in Ceredigion, Wales. Heavy metals discharged from the site are polluting downstream watercourses, leading to widespread Environmental Quality Standards (EQS) compliance failures. Through salt water dilution gauging and water quality sampling, a daily efflux of 876 g of heavy metals was calculated, with contaminant mobilisation occurring mainly in two primary surface streams draining an exposed tailings heap. Electrical resistivity tomography subsurface imaging found a seepage plane within the tailings lagoon wall, whilst the main tailings heap became increasingly saturated with depth. A large adjacent field also had a high potential to convey pollutants in solution, yet its morphological characteristics have limited transmission, as the area acts as a passive treatment type system. With remediation of already polluted water both difficult and expensive, this approach provides a cost-effective way to identify the origins and pathways of contaminants, informing mitigation strategies focussed on containment. Esgair Mwyn is not an isolated case, as abandoned metal mines release at least 860 t of heavy metals annually into UK water bodies. These techniques could reduce or prevent abandoned metal mine hydrological pollution for decades to come, and enable associated UK water bodies to comply with future water quality standards.

## Introduction

There is a rich history of mining in the UK of both ferrous and non-ferrous metals, spanning as far back as the Bronze Age (Mullinger 2004). Output from these mines reached its peak in the nineteenth century due to the high demands of the Industrial Revolution, coupled with major advances in mining techniques (Richardson 1974; Colman and Cooper 2000). However, the twentieth century saw a drastic downturn in demand, concomitantly triggering the decline of market prices within the UK (Mathias 2001). Consequently, hundreds of mines in Wales were abandoned (Johnson 2003). With increased foreign competition and rising extractive costs, the majority of these mines have remained derelict for many years. Unlike coal mining, which was a nationalised industry and therefore developed established standardised regulations, metal mines remained largely privately owned ventures. Consequently, they were often abandoned without any stringent standards to ensure environmentally sound decommissioning took place (Johnston 2004). As a result, numerous adverse consequences have arisen from mine abandonment, not least of which is the contamination of water bodies.

The 2012 *Prioritisation of abandoned non-coal mine impacts on the environment* (Jarvis and Mayes 2012) evaluated the impact abandoned mines are having on aquatic environments of England and Wales, finding approximately 465 water bodies out of 7815 to be affected by pollution from non-coal mines (Mayes et al. 2010). This equates to around 860 t of zinc, lead, cadmium, copper, iron, arsenic and manganese being released into water bodies annually. However, this estimation was based on limited measurements of recognised mine discharges, meaning it is likely to be a gross underestimation of actual values as many diffuse discharges from mines were not included.

This significant contamination has led to widespread failures to comply with the European Water Framework Directive (European Union 2000). Consequently, government agencies such as Natural Resources Wales (NRW) are taking responsibility for identifying and remediating abandoned mines, due to the legislative pressures upon them to ensure the aquatic ecosystems within their jurisdiction meet the necessary European Environmental Quality Standards (EQS) (Table 1).

**Table 1:** Water quality data upstream and downstream of Esgair Mwyn. This data was sourced from historical NRW datasets. Eight samples were collected between 6/7/12 and 23/8/12 in the Gwyddyl. Eight samples were also collected in the Garw and Marchnant between 10/1/12 and 31/5/12. The EQS values presented define the acceptable ‘Annual Average Concentrations’ employed by NRW, as established in WFD (DEFRA, Department for Environment and Rural Affairs 2014). Cu and Pb values are the allowable bioavailable concentrations, with the Zn value also representing the bioavailable concentration, whilst also accounting for ambient background concentrations (ABCs). For the Teifi, this is 2.5 μg/L. The Cd value was established using hardness band 2. Although the datasets do not account for the bioavailable fraction, due to necessary calculation parameters omitted on collection, comparison can still be drawn. It is clear that the concentrations of all the metals increase sizeably downstream of Esgair Mwyn inputs, especially in terms of both Pb and Zn

Sample points	Dissolved metal concentration (μg/L)				
	pH	Pb	Cd	Zn	Cu
Gwyddyl upstream of site	6.86	7	0.1	8	1
Gwyddyl downstream of site	6.95	493	2	558	11
Marchnant upstream of Garw confluence	6.74	2.5	0.1	6	1
Marchnant downstream of Garw confluence	6.29	472	3	839	21
	EQS (μg/L)	1.2	0.08	13.4	1

**Fig. 1:**
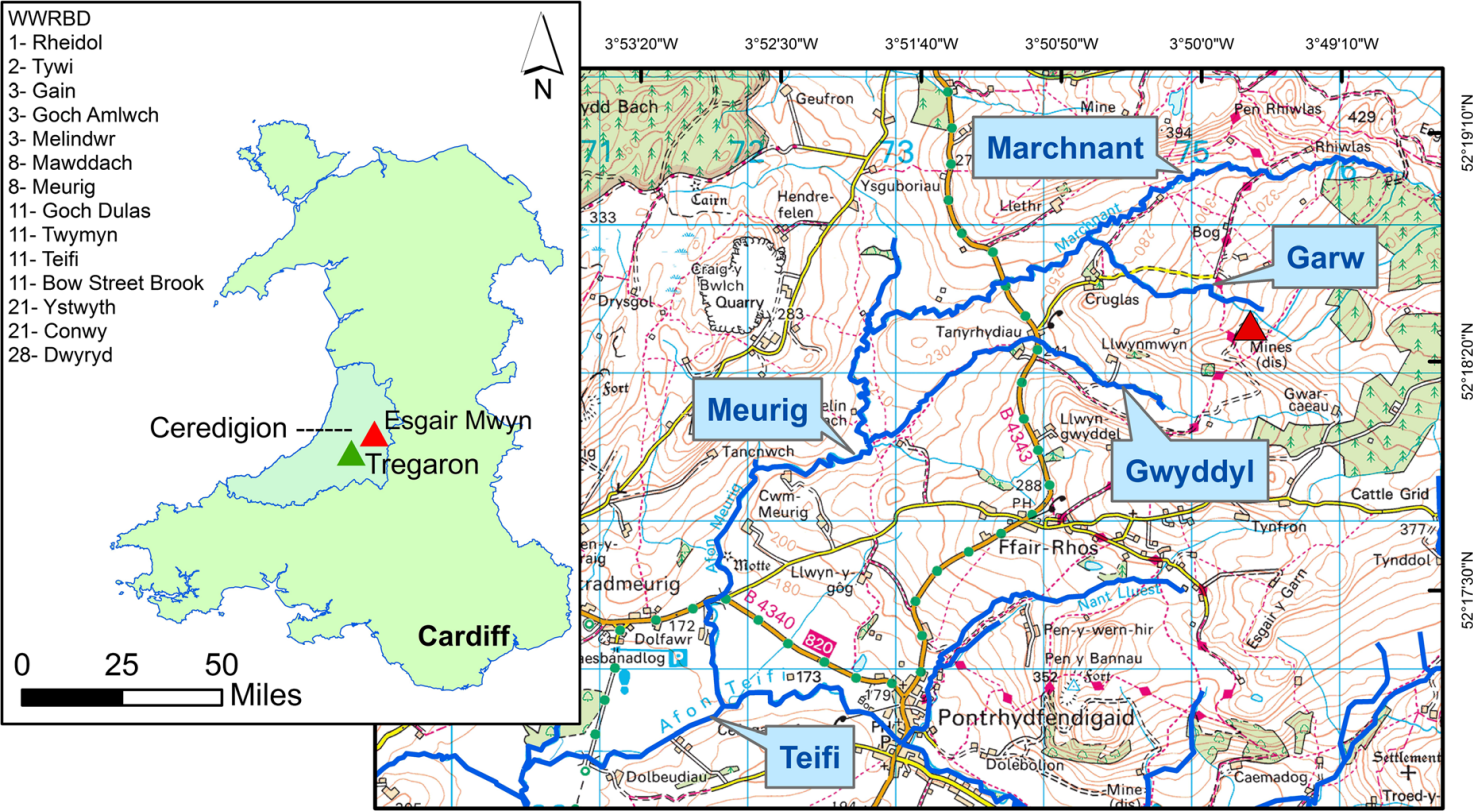
Hydrology. **a** The location of Esgair Mwyn within Wales. The 15 most impacted water bodies located within the WWRBD are also ranked. These represent 50% of the top 30 most impacted water bodies within England and Wales (Mayes et al. 2009). **b** A 1:20,000 map of the area, showing the main rivers associated with the site, as well as a conceptual schematic of how these rivers inter-link with one another. The red triangle denotes the location of Esgair Mwyn

The Western Wales River Basin District (WWRBD) contains numerous high priority contaminated water bodies, with around 12% of rivers suffering from mine-related degradation. Of the top 30 most impacted water bodies in England and Wales, 15 of these are located within the WWRBD (Mayes et al. 2009) (Fig. 1). The Afon Meurig, which is ranked eighth, receives discharge from the Esgair Mwyn mine (Fig. 1).

Previous work has agreed that Esgair Mwyn is responsible for significant water pollution, and is a key contributor to the Afon Meurig and Afon Teifi’s failure to meet necessary EQS, as upstream of the site watercourses comply with EQS heavy metal concentration guidelines (SRK 1997; Parsons Brinkerhoff 2006; Scorey 2012; CH2M Hill 2014; Aecom 2015) (Table 1). As a result, mines such as this have been identified as polluting sites in need of remediation (Environment Agency Wales 2002).

For successful remediation at Esgair Mwyn, a full understanding of the contaminant processes at the site and their interactions with the surrounding environment is crucial. Although dissolved metal concentrations have been intermittently sampled in watercourses of the area since 1980, no integrated surface and subsurface assessment of contaminant sources and transport has been conducted. Flow gauging is a prerequisite for estimations of metal loadings and apportioning sources of contamination, but appropriate data sets for Esgair Mwyn are sparse (Scorey 2012). Salt water dilution gauging (SWDG) is particularly well suited for this purpose (Gees 1990; Kite 1993), although only one SWDG data set is currently available (Aecom 2015). Consequently, the study has conducted extensive flow gauging using SWDG to quantify and apportion metal exports from Esgair Mwyn. Furthermore, little work has addressed the subterranean portion of the site and the role it plays in creating and contributing to contamination, including the crucial hyporheic zone (Gandy et al. 2007).

Electrical resistivity tomography (ERT) is an efficient, non-invasive means of characterising groundwater contamination (Tabbagh et al. 2000; Vereecken et al. 2006; Werth et al. 2010; Fitts 2013; Mao et al. 2015), but has rarely been used in metal mine investigations. Recent work in southern Spain has ascertained that ERT can characterise the spatial extent and grain size characteristics of mine tailings, and reveal the structural composition and stability of tailings ponds. Furthermore, areas of preferential groundwater flow, which have the ability to mobilise and transport contaminants, have been detected and delineated with ERT (Martínez-Pagán et al. 2012; Rey et al. 2013; Martínez-Pagán et al. 2013). According to Archie’s law (Archie 1942), the bulk electrical resistivity of geological media inferred by ERT varies inversely with effective porosity, water saturation and water salinity, and therefore promises to be a powerful tool in delineating and mapping the subsurface hydrological processes within and down flow of Esgair Mwyn.

Therefore, the overall aim of this paper is to introduce how a new integrated assessment of surface and subsurface heavy metal pollution, utilising SWDG, water quality and ERT, can be invaluable in identifying areas of contamination and informing their remediation.

## Study Area

Esgair Mwyn is an abandoned mine in Ceredigion, Mid-Wales, approximately 13 km northeast of Tregaron (52.307914, − 3.828400) (Fig. 1). The mine has been periodically worked since Roman Times, reaching peak-recorded extraction between 1845 and 1913. Around 6402 and 22 t of lead and zinc respectively were removed during this period, leaving around 60,000 t of metal-contaminated fine tailing waste when the mine was closed in 1927. Situated in the Afon Meurig catchment, the site typifies the hundreds of abandoned UK metal mines in that it consists of numerous spoil and tailings heaps, disused shafts, lagoons and abandoned buildings and machinery. Esgair Mwyn lies within the Ceredigion Uplands, a Special Landscape Area (SLA) of national importance in terms of its outstanding ecological and cultural value. Both the Dyfed Archaeological Trust (DAT) and the Royal Commission on the Ancient and Historical Monuments of Wales (RCAHMW) have many Historic Environmental Records (HERs) and National Monument Records (NMRs) on the site, including amongst others the remains of an old smithy. Although none of these features are legally protected, the site is clearly recognised as containing important artefacts of mining heritage. The surrounding area has also been recognised for its ecological importance. Tregaron Bog (Cors Caron) is a Ramsar wetland site and National Nature Reserve (NNR), and the Afon Teifi is a designated Special Area of Conservation (SAC), both of which lie downstream of Esgair Mwyn. However, the surrounding poorly drained moorland only supports agriculture and rough grazing as its main land use.

A dendritic network of surface channels can be seen, with northern areas of the site drained by the Nant y Garw, which flows north-westwards into the Afon Marchnant (Fig. 1). The southern portion of the site drains into the Nant y Cwm Gwyddyl, which in turn flows west into the Afon Marchnant, subsequently becoming the Afon Meurig. The Meurig then flows 6 km south-westerly, where it constitutes a main tributary of the Afon Teifi. Metal contamination of this SAC is therefore of critical concern.

Esgair Mwyn lies upon Devil’s Bridge strata, a turbiditic formation of interbedded hemipelagic marine mudstones and sandstones (Schofield et al. 2009), overlain by Quaternary Till deposits. The local mineral lode runs directly through the site, striking approximately WSW/ENE. Several shafts, levels and an adit provided access to the lode, and these subsurface mine workings may provide preferential hydrological pathways for subterranean flows. These flows discharge at various points at the surface, such as a spring at the western base of the main tailings heap, thus supplying surface waters with heavy metals that have been mobilised and transported beneath the surface (Parsons Brinkerhoff 2006). Furthermore, precipitation falls upon the surface of tailings and soil at the site, and percolates downwards. As the natural hydraulic gradients under which subsurface water flows usually mirror surface topography, this groundwater will flow from the high hydraulic heads which exist at the top of the east-west trending ridge, to the low hydraulic heads to the north or south of the ridge (Fitts 2013). Therefore, as groundwater and surface water flows are usually in the same direction as a direct outcome of topography, we can conclude that groundwater flow at Esgair Mwyn is probably also in a north-westerly direction. However, again, the presence of subterranean workings may artificially lower the potentiometric surface in localised areas due to drawdown, meaning the hydrogeology of the site is likely to be complex (Parsons Brinkerhoff 2006).

## Materials and Methods

**Fig. 2:**
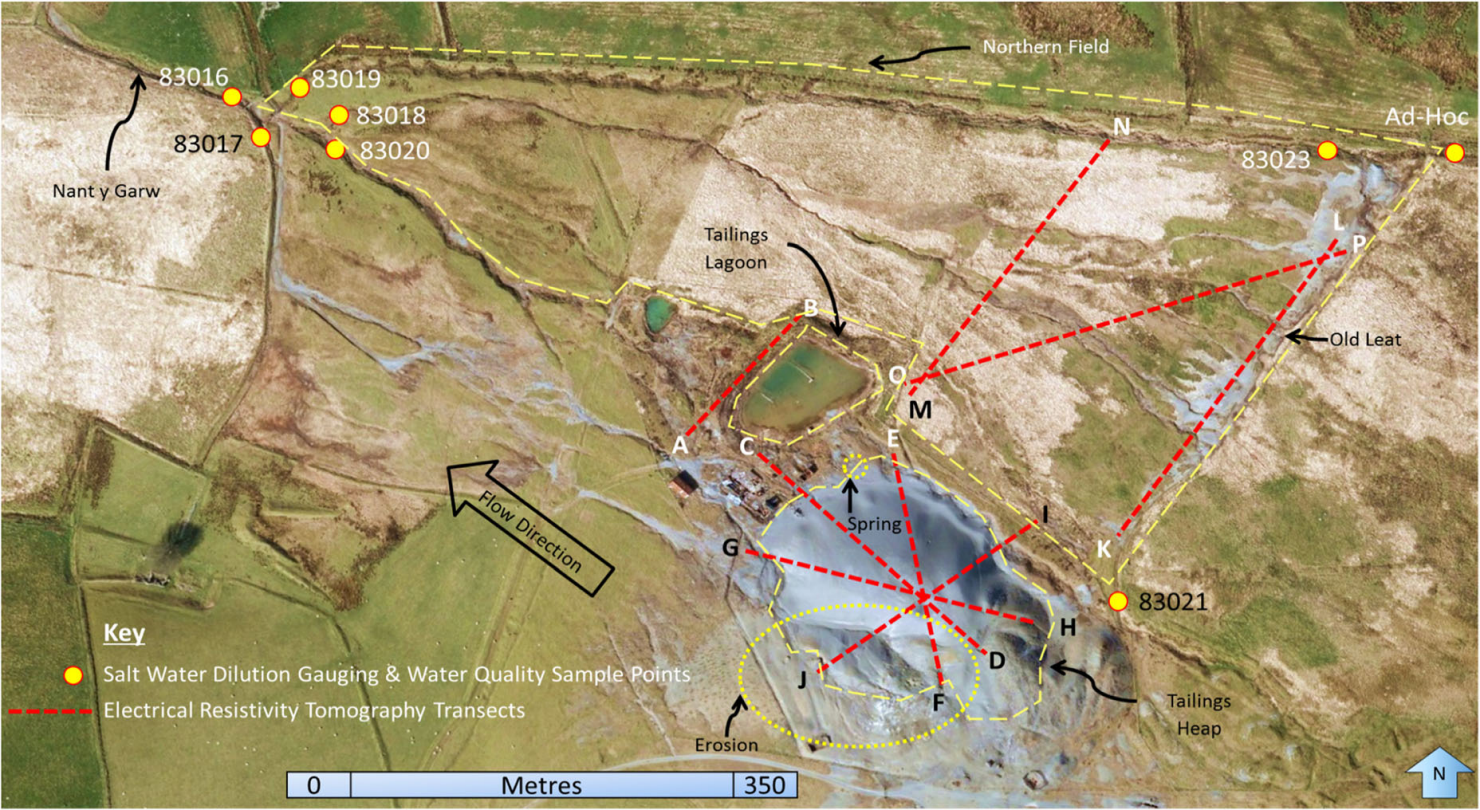
Sampling locations. The flow gauging, water quality and ERT sampling points are detailed, investigating the main areas of interest: the main tailings heap, tailings lagoon and northern field

Flow gauging and water quality sampling points were established, based on pre-existing locations that have been sporadically monitored since 1980 by NRW and its predecessor bodies (Fig. 2). Seven of these were selected for use, chosen as representative of the main surface hydrological pathways, and each revisited once in July, August and September 2015. Samples were taken during baseflow conditions rather than stormflow, to be more representative of normal values. The relative method of dilution gauging developed by Littlewood (1987) was adopted. This entails filling two buckets with stream water, with one to be used for a bankside calibration test and the other to be mixed with 30 g of salt to create the salty injection solution. In a single-gulp application, a known volume of this salty solution is introduced to the stream, with an electrical conductivity meter placed at least 25 m downstream where adequate mixing will have occurred (Butterworth et al. 2000). Electrical conductivity is recorded at 10-s intervals until a return to base values is observed. Through this, a chemograph response denoting initial plume arrival, peak concentration, along with return to base values was identified for each sampling location, allowing the discharge (*Q*) to be calculated. Once the *Q* of the main surface hydrological pathways was established, it was combined with water quality regarding metal content. These water quality data were derived from extracting a total metal sample (TMS) and filtered metal sample (FMS) from water samples from each stream on each of the three sampling visits, with this analysis being performed at NRW’s UKAS-accredited laboratory facility. The combined heavy metal export of all surface streams was subsequently quantified, with the additions each main stream made to this total apportioned.

The acquisition of ERT data involved collecting series of quadripole measurements with an IRIS *Syscal Pro* imaging system, with 24 electrodes placed equidistantly at 5-m intervals along a linear profile at the ground surface (4 m for transect AB). Two pairs of stainless steel electrodes are used for the injection of an electrical current and the measurement of the resulting electrical voltage, respectively, where electrode spacing and thus depth penetration is increased sequentially (Reynolds 2011). Known as the Wenner-α array, this electrode arrangement is less prone to noise and more sensitive to horizontal variations in bulk resistivity than potential alternatives (Dahlin and Zhou 2004; Samouëlian et al. 2005). The height of each electrode location was measured using an Abney level and distance measurements.

Using Ohm’s law together with an array geometry factor, these data are then converted to an apparent resistivity before being tomographically inverted to produce 2-D images of bulk resistivity in the subsurface. Inversion used the finite-difference code DCIP2D (Oldenburg and Li 1994; Li and Oldenburg 2000) with 15 data levels, and an average of 7 iterations required to produce final inverted ERT models that fitted the data within a 5% root-mean-square (RMS) error level.

Adopting a previously developed best practice approach (Thompson et al. 2012, 2017), measurement uncertainty was quantified in two main ways. First, each quadripole measurement was repeated with current and voltage electrodes interchanged, thus creating a direct and a reciprocal datum for each quadripole. Second, the majority of our ERT profiles had cross-over points where bulk resistivities should be equal. In both cases, the standard deviation between measurements can serve as a valuable indicator of uncertainty (Thompson et al. 2012, 2017).

ERT survey locations focused on the tailings lagoon wall, the main tailings heap and the northern field (Fig. 2). Several leakage events have been noted in historical site reports, where tailings waters highly concentrated with heavy metals have diffusely escaped through the lagoon wall, instead of from the designated discharge point. Consequently, this was a key area of focus as loaded waters from within the lagoon are failing to be properly redirected to the recirculation and emergency lagoons as was originally designed. Additionally, the main tailings heap and northern field are the major areas in which tailings have been deposited at the surface, and therefore pose the largest potential hydrological contamination risk. As the lagoon wall was only 50 m long, a single resistivity line running atop the bund into adjacent runoff streams was sufficient to investigate it (Fig. 2). The main tailings heap is far more extensive; therefore, it was covered by several transects between 100 and 120 m in length to garner a representative coverage of the subsurface. Thus, four ERT profiles with variable azimuths about a central cross-over point were used to sample the tailings heap (Fig. 2). This point was situated on a conical depression at the top of the heap, about 2 m in depth and suspected to facilitate a preferential flow pathway for precipitation through the tailings.

The north field was investigated by three transects, each 120 m long (Fig. 2). The first ran along the top of the old leat, encompassing the start of numerous incised surface runoff channels as well as deposited tailings. One hundred metres downstream, a second profile was taken parallel to the first. This provided good spatial coverage of the area, with the main surface hydrological pathways being investigated at various channel stages. A third transect then ran diagonally connecting the two.

## Results

### Salt Water Dilution Gauging and Water Quality Results

**Fig. 3:**
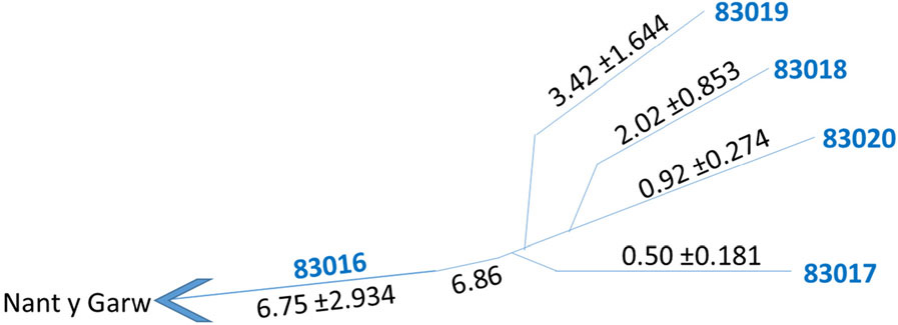
Combined flow rates of the four main streams converging into 83016. All values are in litres/second. On average, only 0.11 L/s of flow is unaccounted for, with standard errors also displayed

**Fig. 4:**
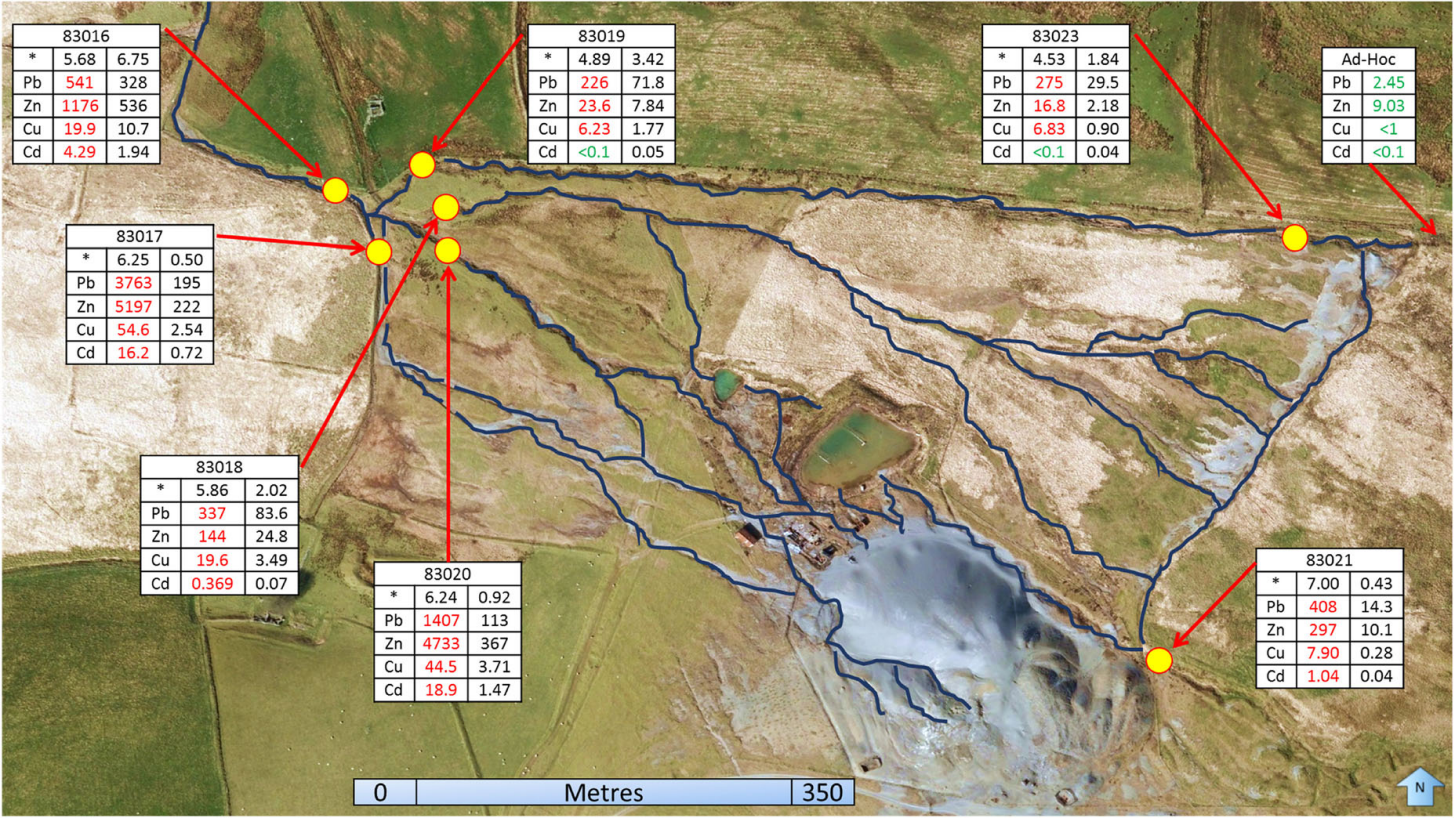
Dissolved metal concentrations and loadings. Column 1 denotes the heavy metals tested for through water quality. Column 2 displays dissolved metal concentrations (μg/L), with values in red denoting EQS exceedance, whilst values in green comply with EQS (Table 1). Column 3 shows dissolved metal loadings (g/day), with a total of 877 g of heavy metals exported from the site in the Nant y Garw (83016) daily. The asterisk row denotes firstly the flow rate (L/s), followed by stream pH value. The flow rate is an average value based on 4 monthly measurements performed by SWDG. The dendritic nature of surface channels is also displayed

Individual stream discharges were calculated for each of the three sampling visits and combined with Aecom (2015) flow gauging to produce average flow rates for each sampling location. On each occasion, the cumulative flow of the four main surface streams that drain the site to form the Nant y Garw equated well to the flow rate measured in the Nant y Garw downstream of their confluence (Fig. 3). Stream segment 83019 had the greatest flow rate on each occasion. Average conductivity values showed streams 83020 and 83017 had the highest values at 152.8 and 125.5 μScm^−1^, respectively, with significantly lower values of 66.4, 61.5, 55.8, 40.9 and 32.8μScm^−1^ seen in streams 83016, 83023, 83019, 83021 and 83018 respectively. When the total and dissolved concentrations for each of the tested metals in the main outlet stream (83016) were compared, it showed an average of 80% of the total metal leaving the site is dissolved. All streams were found to have high total metal concentrations, especially of lead (261–2553 μg/l) and zinc (25–5153 μg/l), with values exceeding EQS in all cases, except for 83019 and 83023 cadmium values (Fig. 4). Upstream of sample point 83023, before the stream had received any input from the mine, metal concentrations of the sample taken were all compliant with EQS (Fig. 4).

**Fig. 5:**
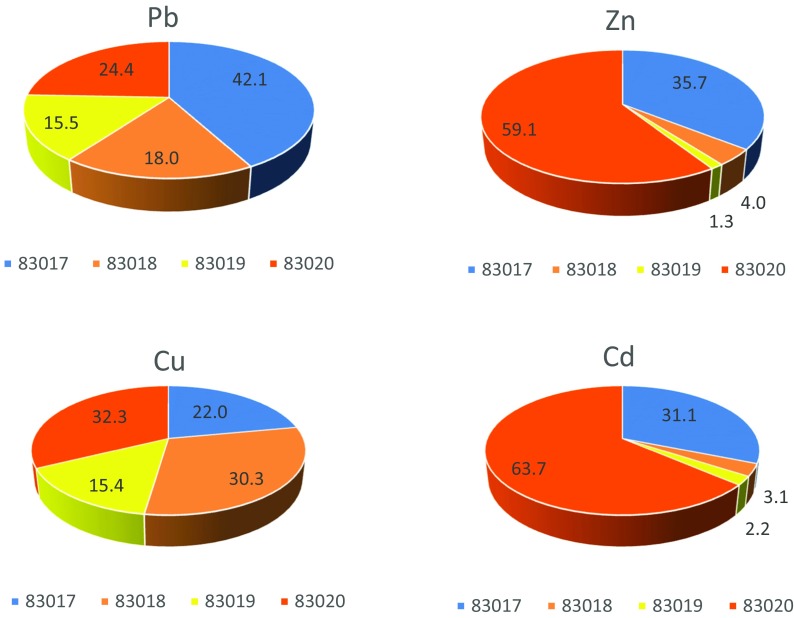
Apportioned heavy metal loadings of the four main streams. Values were calculated from average dissolved daily loadings. There was 136 g/day excess Pb in the four main streams than in 83016’s export, 86 g/day of Zn, 0.8 g/day Cu and 0.4 g/day Cd

Dissolved metal concentrations were then combined with flow gauging data to produce dissolved metal loadings (Fig. 4). Average daily loadings of 328 g lead (Pb), 536 g zinc (Zn), 10.7 g copper (Cu) and 1.94 g cadmium (Cd) discharged into downstream watercourses. These daily loadings were then apportioned with respect to each stream’s contribution (Fig. 5). Of the 463 g/day of lead, 42% was derived from 83017 and 24% from 83020, with the remaining two streams providing similar inputs (16 and 18%). Of the 621 g/day of zinc measured, 83020 was by far the largest contributor with almost 60%. 83017 supplied most of the rest (36%), whilst contributions from 83018 and 83019 were negligible at 4 and 1.3% respectively.

Stream contributions to the 11.7 g/day of copper were relatively balanced, but with 83020 again delivering the greatest proportion. Of the 2.3 g/day of cadmium measured, 64% was supplied by 83020. As with zinc, 83017 provided most of the remaining cadmium (31%), with 83018 and 83019 making minor contributions (3.1 and 2.2% respectively). Thus, in all cases except lead, 83020 makes the largest contributions to the overall metals export from the site. Conversely, stream 83019 consistently supplies the lowest proportion of each of the heavy metals measured (Fig. 5).

Far lower metal loadings were seen in the streams draining the north field. In the stream sampled at 83019 and 83023, an increase of over 50% in lead, zinc and copper values between the two sampling points was measured, indicating a significant contamination source between these two points (Figs. 2 and 4). Cadmium values on the other hand were more similar between the two sampling points.

### Electrical Resistivity Tomography Results

Each ERT profile was subjected to the two types of error analysis. First, the standard deviation about the mean of the difference between each quadripole direct and reciprocal resistivity values was calculated, yielding percentage error values respectively of 6.2 and 6.6% for the spoil heap and north field. Any values exceeding one standard deviation were then excluded, as reciprocity is a good indicator of error (Thompson et al. 2012). A total of 48 quadripole readings or 8% of the 590 quadripoles in total were thus removed.

The cross-over point analysis of the tailings heap, where profiles CD, EF and GH overlapped (Fig. 2), had a mean deviation of 854 ± 53.3 Ω m. The two cross-over points of the north field, where transects KL and OP intersected, as well as OP and MN, had a mean deviation of 253 ± 16.67 Ω m. These deviations about the mean of 53.3 and 16.7 Ω m were deemed acceptable and were likely due to small random errors introduced by the equipment or poor electrode-subsurface contact (Thompson et al. 2012), meaning no further corrections were therefore applied.

**Fig. 6:**
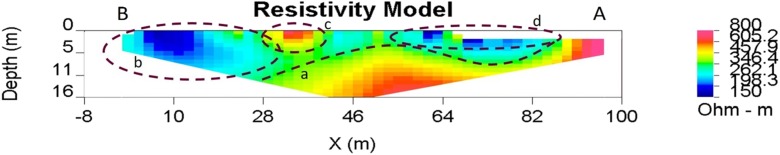
Tailings lagoon wall ERT profile. Transect AB has a dual layer structure from 8- to 11-m depth (a), with resistivity increasing with depth. Surface overland flow channels correlate to low resistivity zones (d), with a localised area of high resistivity around 34 m where a plastic lagoon overflow pipe is buried (c). A large zone of lower resistivity at around 10 m along the profile coincides with the old channel bed over which the lagoon was extended (b)

The wall of the lagoon has a dual layer structure (Fig. 6), with values respectively lower and much greater than ~ 300 Ω m in the upper and lower layers, separated by a transition zone at depths of 8–11 m. This dual layer structure and the localised areas of anomalous resistivity imprinted on it, such as the low and high resistivity anomalies at the surface and at respective distances of 10 and 34 m along the profile, are interpreted in an integrated context in Sections 5.1 and 5.2.

**Fig. 7:**
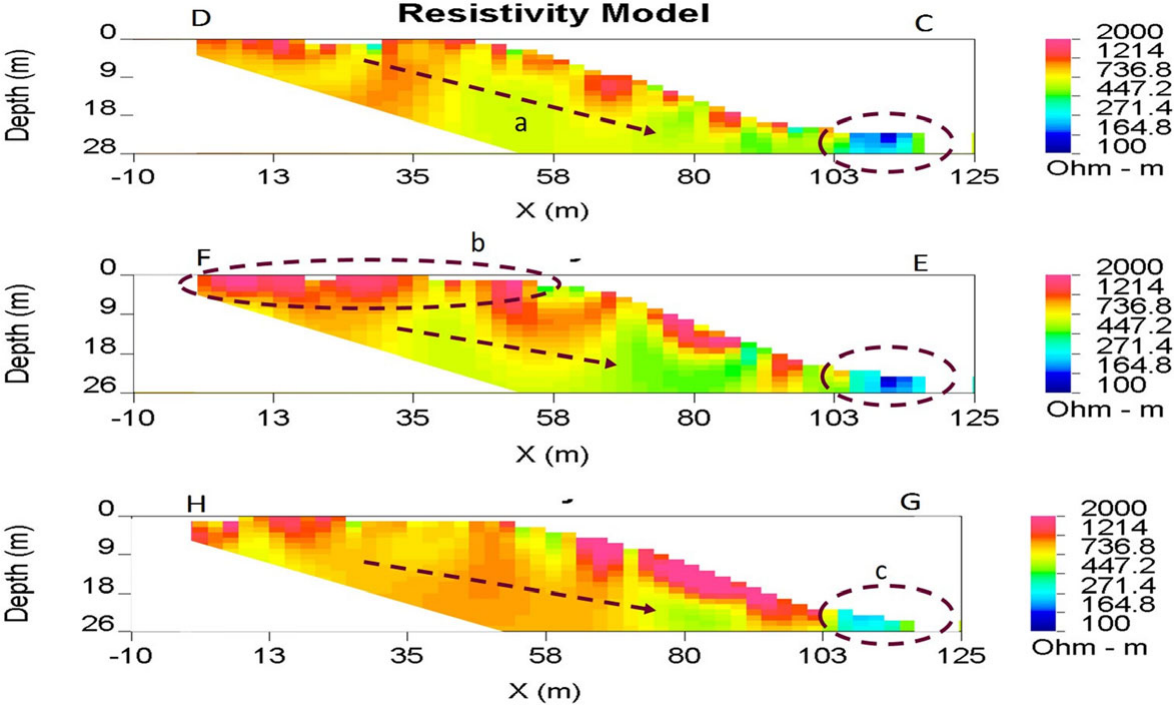
Tailings heap ERT profile. Clear lineation of higher resistivity surface at around 2000 Ω m (b), with resistivity decreasing with depth to around 400 Ω m (a). Toward the periphery of the tailings heap, an area of low resistivity is seen in all cases, coincident with surface streams and water-logged conditions around the circumference of the tailings (c)

The three profiles investigating the subsurface of the main tailings heap are shown in Fig. 7. Although each varies to some degree, lineation of a higher resistivity layer is discernible near the surface at around 2000 Ω m, with resistivities decreasing to values of around 500 Ω m at depth. Observations of the ground surface of the tailings heap slopes found looser, drier material at the surface, which became finer, more compacted and wetted beneath the top 10–15 cm. There is a general decrease in resistivity from the upper to the lower end of each profile, with values changing from > 2000 Ω m at the top end of the tailings heap, to ~ 700 Ω m in the middle and < 400 Ω m toward the bottom. At the ends of profiles C, E and G at the heap’s circumference (Fig. 2), an area of low resistivity can be found (highlighted in Fig. 7). This coincides in all cases with the location of a surface stream and related water-logged ground conditions.

**Fig. 8:**
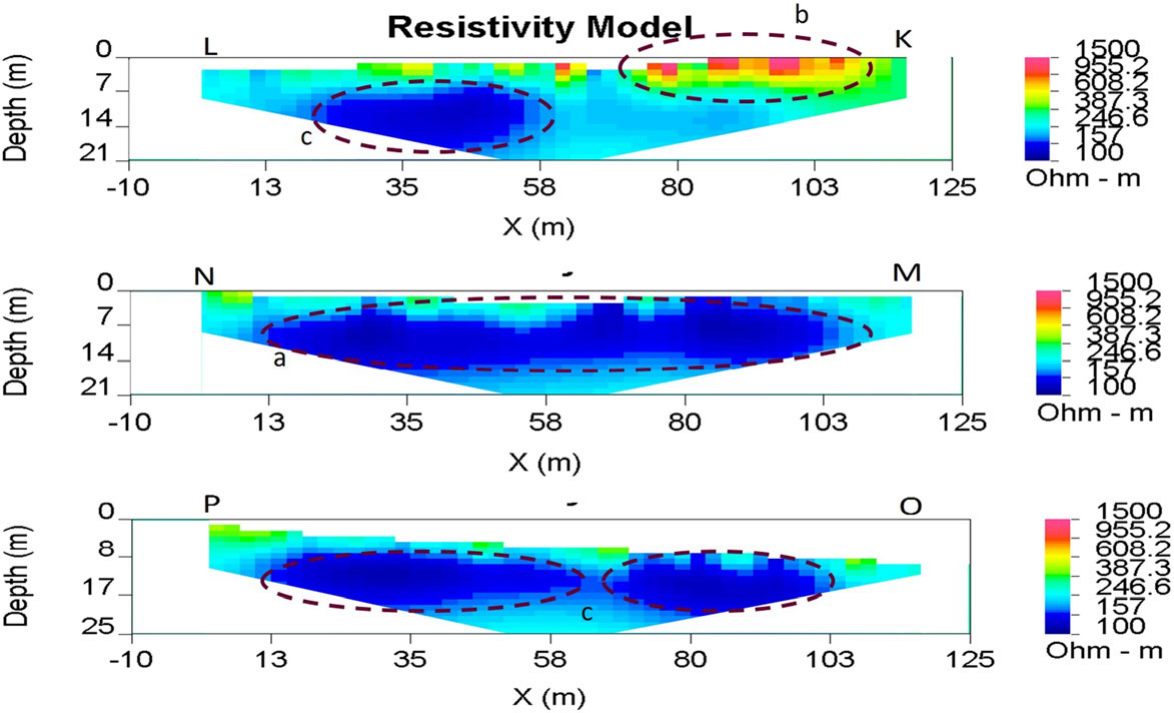
North field ERT profile. The transects are dominated by low resistivity material around 100–250 Ω m, with areas < 100 Ω m resistivity seen in abundance between ~ 7- and 18-m depth (a and c). Some anomalously high resistivities are seen at the surface of transect KL, coinciding with areas of compact and dense tailings deposited along the old leat (b)

Transects in the north field are dominated by low resistivity material of less than 250 Ω m (Fig. 8). The only exceptions are the elevated resistivities at end K of profile KL which coincide with areas of compact ground and dense deposited tailings. Otherwise, resistivity declines beneath the surface until a layer with resistivities much less than 100 Ω m begins at around 3–4-m depth. This low resistivity zone extends laterally across profiles MN and OP, and half of profile KL (Fig. 8).

## Discussion

### Surface Hydrological Contamination

Comparisons between metal concentrations and established EQS show that Esgair Mwyn represents a major source of contamination to downstream watercourses, with an average of 876 g of heavy metals being discharged into the Nant y Garw on a daily basis (Fig. 4). As a consequence, the Afon Meurig into which the Garw flows is currently failing under the Water Framework Directive (Table 1 and Fig. 1), especially in terms of lead and zinc levels. Although the four primary surface drainage routes at the site all contribute to the overall loadings seen within the Garw, apportionment has revealed that some are transporting larger quantities of pollutants than others.

The stream sampled at 83020 appears to be the most problematic discharge, as it is the largest contributor to overall loadings. The stream receives input via surface overland flow from the south side of the main tailings heap, which bypasses the tailings lagoon as it flows north-westward (Fig. 4). Heavily incised channels on the south side of the tailings heap indicate erosion (Fig. 2), with water from them becoming enriched with metal sulphates from the tailings, either in solution or in particulate form (Navarro et al. 2008). The stream also receives water which discharges from the tailings lagoon via designated outflow pipes, as well as the recirculation and emergency lagoons (Fig. 2). The tailings lagoon collects drainage from several flows which all come into contact with the main tailings heap, meaning it will inevitably contain water enriched with heavy metals (Sidle et al. 1991). The lagoon was designed to capture and store water, allowing the contaminants within to settle out. However, after reprocessing of the tailings was granted, the lagoon had to be expanded in order to increase its storage capacity (Wardell Armstrong 1988). As a result, in 1974, the lagoon bund height was increased via the addition of stony clay material from the surrounding area, which differed from the top-soil and tailings media comprising the existing wall (Hydrotechnica 1987). Moreover, the lagoon was also extended northwards in 1977 over a former stream channel bed. The change in lithology coupled with the presence of an old channel bed is conjectured to have created a preferential seepage plane at the north end of the lagoon bund, thus allowing water within the lagoon containing highly concentrated heavy metals to flow diffusely downstream. The ERT survey of the lagoon wall appears to support this theory, bringing into question whether this seepage plane may also lead to structural instabilities developing within the wall, which could result in a catastrophic failure in the future.

The stream sampled at 83017 was the second largest contributor to the Nant y Garw’s metal loadings (Figs. 2 and 5). Like 83020, this stream also receives runoff directly from waters flowing down the south side of the tailings heap, thus enabling metals, especially lead and zinc, to be readily picked up and transported in solution. Both this stream and that sampled at 83020 have far greater concentrations of lead and zinc than 83018 and 83019 due to the large, metal-rich erosional surface they are draining, resulting in higher metal loadings despite lower flow rates than 83018 and 83019. The two streams also have significantly higher background conductivities than any of the other streams, again indicating that they are both heavily contaminated.

The two streams sampled at 83018 and 83019 make lower metal contributions to the Nant y Garw (Figs. 2, 4 and 5), despite having lower pH values than streams 83017 and 83020. Lower stream pH value decreases the adsorption capabilities of particles in solution, facilitating increased kinesis of heavy metals within watercourses (Li et al*.* 2013). There are a number of reasons that might explain these smaller loadings. 83018 and 83019 both bypass the main tailings heap, only coming into contact with tailings that are deposited (and not subject to active erosion) along the top of the old leat (Fig. 4). In addition, the two streams receive water mainly from the north field (Fig. 4), which has a thicker soil substrate (Cranfield 2015), and greater vegetation cover from grasses as well as peaty moss. Although the ERT surveys of the north field indicate that it is highly saturated, it is conjectured that this area may actually be acting as a type of wetland, allowing for the attenuation of metals in the soil (Mungur et al. 1997; Matagi et al. 1998; Sheoran and Sheoran 2006), thus limiting the metal loadings seen at points 83018 and 83019. Peaty moss has been found to be very effective at binding metals, due to its large surface area and the presence of lignin and cellulose (Babel and Kurniawan 2003). Therefore, it is speculated that these streams contribute less heavy metal pollutants to the Garw due to their lower exposure to metal-rich tailings, and because the peaty subsurface of the north field is currently acting as a heavy metal sink.

### Subsurface Hydrological Contamination

The expansion of the tailings lagoon was accomplished in part by adding material to the top of the pre-existing bund (Fig. 2). The material added to increase the bund height comprised stony clay from the area surrounding the lagoon, which differed from the top-soil and tailings material comprising the existing wall (Hydrotechnica 1987). This lithological discontinuity could explain the dual layer structure seen within the resistivity profile of transect AB (Fig. 6). The clay-bearing material added to the top of the pre-existing lagoon bund is finer-grained than that beneath it, meaning it has smaller pore spaces correlating to a large surface area (Doerr et al. 2000). Consequently, this material will have stronger capillary forces attracting water molecules to the clay mineral surfaces, whilst its flocculated card-house structure implies low effective porosity and therefore poor water drainage. Clay mineral-rich media therefore tend to have anomalously low bulk resistivities (Daily et al. 1992) (Fig. 6). The layer beneath has a lower clay content and therefore lower water retention capabilities, and thus higher resistivities (Rhoades et al. 1976; Kalinski and Kelly 1993; Abu-Hassanein et al. 1996; Michot et al. 2003) (Fig. 6).

The low resistivity anomaly dominant at distances around 10 m along the profile (Fig. 6) may be indicative of the preferential seepage plane. This area coincides with the location of an old channel bed, and this pre-existing flow pathway, coupled with the change in lithology, implies that this area is probably more saturated than other areas of the bund. This may therefore explain why seepage is visible here when the lagoon water level is high (Wardell Armstrong 1988), which in turn may be contributing in part to stream 83020’s high metal loadings. In addition, the localised areas of higher resistivity at distances of 34 and 52 m (Fig. 6) coincide with the locations in which two plastic outflow pipes are buried, which will act as insulators.

The areas of low resistivity around the circumference of the tailings heap (Fig. 7) are consistent with the presence of areas of overland flow and water-logged conditions which, in all cases, facilitate the conductance of electrical currents (Tabbagh et al. 2000). Higher resistivities observed abundantly close to the surface of the tailings heap (Fig. 7) can be explained by surface material that is less compacted than at depth and of lower water content (Samouëlian et al. 2005). This certainly agrees with the high resistivity values recorded at the top of transect EF, where large, loose, dry rocks originating from shaft excavation can be found. The decrease in resistivity observed with depth may therefore be caused in part by materials becoming more compacted, with the overburdening pressure increasing proportionally with depth from the surface (Weller 1959; Skempton 1969). The more wetted finer-grained tailings observed beneath the top 10–15 cm of the surface may also indicate that saturation increases with depth, contributing to the decreasing resistivity gradient seen in all profiles (Fig. 7). With rainfall percolating through the profile and wetting tailings at depth, there is also a high contaminant mobilisation potential within the tailings heap (Schwartz et al. 2008), especially in terms of acid mine drainage (AMD). When the mine was abandoned, the tailings were left exposed to both water and oxygen, facilitating oxidation of any pyrite within. Any resultant AMD produced would eventually be flushed out into receiving surface streams 83020 and 83017 where it would dissolve metal compounds, further mobilising contaminants which contribute to the overall heavy metal export from Esgair Mwyn.

The ERT profiles of the north field are unanimously dominated by low resistivities that extend laterally across the entire field (Fig. 8). These may be indicative of saturated conditions beneath the surface (Zhou et al. 2001), as they are consistent with water-logged conditions observed at the surface. Such conditions facilitate contaminant transport and thus can explain the 50% increase in metal loadings seen between water quality sampling points 83023 and 83019 (Fig. 8).

The data generated from ERT have been useful in making tentative inferences about the spoil heap and north field, as well as the structural make-up and integrity, or lack thereof, of the lagoon wall at Esgair Mwyn. This cost-effective technique can be applied in similar situations elsewhere, with results potentially allowing for targeted management strategies to be put in place without expensive and potentially destructive earthworks having to be conducted. However, it is recommended that any hypothetical inferences like the ones deduced above be ground-truthed by future investigations.

### Possible Remediation Measures

This study has highlighted the main areas of the Esgair Mwyn site from which pollution is arising, and thus, the areas which should be the focus of future remediation projects. The major surface hydrological contamination pathways appear to be the streams sampled by 83020 and 83017, which transport water which has come into direct contact with the tailings heap. Therefore, limiting the exposure of these surface flows to the main tailings or treating the contaminated flows could ameliorate the high metal loadings currently seen in these two streams. Possible remediation ideas to facilitate this could be:

- Channelization: As there are numerous shallow and braided surface runoff channels that flow over or adjacent to the main tailings heap and contribute to these two streams, channelization could concentrate such flows together, and divert their contents into the tailings lagoon to facilitate the settling out of contaminants. These channels would benefit from being lined with resistant impermeable material, in order to reduce erosion and make clearing of tailings build-ups simpler (Kuyucak 2002). However, our ERT profiles have shown that the structural integrity of lagoon bunds should be investigated further to ensure no release of contaminants would occur if flows were diverted there.
- Capping the tailings: ERT results pertaining to the main tailings heap have indicated that water is percolating through the tailings heap causing increased saturation with depth and subsequent water discharge at the surface through features such as the spring at the west end of the tailings heap. Capping the heap would: (a) prevent the infiltration of water; (b) reduce oxygen availability to the heap; and (c) prevent surface runoff flowing over the heap from mobilising heavy metals (Akcil and Koldas 2006). Materials successfully used to cap tailings heaps include clay (Gandy and Younger 2003) and synthetic liners (Haug and Pauls 2002). The nearby Frongoch mine is a prime example of where NRW have been able to remediate hydrological pollution using such liners. However, for this strategy to be feasible at Esgair Mwyn, the tailings heap would need to be re-sized and regraded, as it is currently too large and steep.
- Treatment system: Various active and passive treatment systems can be used to treat contaminated minewater discharges. Passive systems, such as vertical flow ponds (VFP), which are sulphate-reducing bioreactors, could be implemented to remove the heavy metals contained within the site discharges (Johnson and Hallberg 2005). The Force Crag Mine in the Lake District has seen the successful installation of a passive mine water treatment system, where two VFP lined with a resistant membrane and topped with a compost treatment mix allow diverted water to filter out heavy metals. Reductions of over 94% in Pb, Zn and Cd have resulted from the scheme, highlighting how effective such a system could be if installed at Esgair Mwyn (Edwards 2017, *pers comms.*).
- Wetland establishment: As the north field has been inferred to be acting as a wetland, flows originating in the north east and south east of the site could be diverted here and a wetland system encouraged (Younger 2000; Kadlec and Wallace 2008).

Recently, NRW has commissioned Aecom to produce a design proposal for remediation at the site. The ideal design would involve constructing a surface water management system for Esgair Mwyn in order to better control and direct surface flows away from areas of high contamination potential. The aim of such a system would be to reduce the volume and loadings of metals within the surface streams, thus reducing the size of any future treatment system that might need to be installed.

## Conclusion

From the water quality, flow gauging and resistivity imaging conducted within this study, it is concluded that Esgair Mwyn represents a major contamination source contributing to the failure of downstream watercourses in meeting WFD objectives. Streams sampled at 83020 and 83017 provide the greatest proportions to overall lead, zinc and cadmium loadings (Fig. 5), with all streams inputting relatively equal amounts of copper into the Nant y Garw. In addition, there is strong evidence to suggest that the extension and enlargement of the lagoon and its bund in 1974 and 1977 have facilitated the unregulated seepage of contaminated water into surface flows, without first settling out in the recirculation and emergency lagoons as intended. The tailings heap is surmised to become saturated with depth, subsequently loading the streams which drain it with large proportions of heavy metals. The north field has been shown to be water-logged, and is consequently capable of mobilising substantial quantities of heavy metals in solution. However, subsurface morphological characteristics may restrict the transportation of contaminants somewhat, as the area is functioning as a wetland passive treatment type system. Therefore, these areas have a high potential to mobilise heavy metals within the subsurface hydrology, yet the subterranean conveyance of contaminants may be limited to some degree. Various approaches to remediation are available to ameliorate the problematic surface and subsurface hydrological contamination occurring at the site. Future studies in support of remediation measures at Esgair Mwyn could include extended coverage of ERT profiles with longer electrode arrays for increased depth penetration, and the use of access holes to better characterise subsurface contaminant conditions and transport. The technique used within this study can be considered as good-practice, providing a general overview of both surface and subsurface contaminant mobilisation and transport. With Esgair Mwyn being just one of hundreds of abandoned metal mines in the UK, the practices used here could be employed at other sites to help inform mitigation and remediation strategies for the future. Indeed, NRW has already begun utilising SWDG more readily when stream gauging polluted mine water discharges.

The techniques used within this study can be considered as good-practice, providing a comprehensive overview of both surface and subsurface contaminant mobilisation and transport. With Esgair Mwyn being just one of hundreds of abandoned metal mines in the UK, the practices used here could be employed at other sites to help inform mitigation and remediation strategies for the future.

## References

[CR1] Abu-Hassanein ZS, Benson CH, Blotz LR (1996). Electrical resistivity of compacted clays. Journal of Geotechnical Engineering.

[CR2] Aecom. (2015). *Teifi metal mines project: Esgair Mwyn remedial options appraisa*l*,* Aecom, Cardiff, 47073560.

[CR3] Akcil A, Koldas S (2006). Acid mine drainage (AMD): causes, treatment and case studies. Journal of Cleaner Production.

[CR4] Archie GE (1942). The electrical resistivity log as an aid in determining some reservoir characteristics. Transactions of the AIME.

[CR5] Babel S, Kurniawan TA (2003). Low-cost adsorbents for heavy metals uptake from contaminated water: a review. Journal of Hazardous Materials.

[CR6] Butterworth JA, Hewitt EJ, McCartney MP (2000). Discharge measurement using portable dilution gauging flowmeters. Water and Environment Journal.

[CR7] CH2M Hill (2014). Land availability survey for metal mine treatment systems.

[CR8] Colman TB, Cooper DC (2000). Exploration for metalliferous and related minerals in Britain: A guide.

[CR9] Cranfield University (2015). Soilscapes: LandIS Soil Portal.

[CR10] Dahlin T, Zhou B (2004). A numerical comparison of 2D resistivity imaging with 10 electrode arrays. Geophysical Prospecting.

[CR11] Daily W, Ramirez A, LaBrecque D, Nitao J (1992). Electrical resistivity tomography of vadose water movement. Water Resources Research.

[CR12] DEFRA, Department for Environment & Rural Affairs (2014). Water Framework Directive implementation in England and Wales: New and updated standards to protect the water environment.

[CR13] Doerr SH, Shakesby RA, Walsh R (2000). Soil water repellency: its causes, characteristics and hydro-geomorphological significance. Earth-Science Reviews.

[CR14] Environment Agency Wales (2002). Metal mines strategy for Wales.

[CR15] European Union (2000). Directive 2000/60/EC of the European Parliament and of the Council establishing a framework for the Community action in the field of water policy.

[CR16] Fitts CR (2013). Groundwater science.

[CR17] Gandy CJ, Younger PL (2003). Effect of a clay cap on oxidation of pyrite within mine tailings. Quarterly Journal of Engineering Geology and Hydrogeology.

[CR18] Gandy CJ, Smith JWN, Jarvis AP (2007). Attenuation of mining-derived pollutants in the hyporheic zone: a review. Science of the Total Environment.

[CR19] Gees, A. (1990). Flow measurement under difficult measuring conditions: field experience with the salt dilution method. In *Hydrology in Mountainous Regions I. Hydrological measurements: The water cycle* (Vol. 193, pp. 255–262). IAHS Publications.

[CR20] Haug MD, Pauls G (2002). A review of non-traditional dry covers.

[CR21] Hydrotechnica (1987). Mine tailings project: Esgairmwyn.

[CR22] Jarvis, A. P., & Mayes, W. M. (2012) Prioritisation of abandoned non-coal mine impacts on the environment: (SC030136/R2) the national picture*.* Environment Agency, Bristol.

[CR23] Johnson DB (2003). Chemical and microbiological characteristics of mineral tailings and drainage waters at abandoned coal and metal mines. Water, Air, and Soil Pollution: Focus.

[CR24] Johnson DB, Hallberg KB (2005). Acid mine drainage remediation options: a review. Science of the Total Environment.

[CR25] Johnston D, Jarvis AP, Dudgeon BA, Younger PL (2004). A metal mines strategy for Wales. International Mine Water Association Symposium 1.

[CR26] Kadlec RH, Wallace S (2008). Treatment wetlands.

[CR27] Kalinski RJ, Kelly WE (1993). Estimating water content of soils from electrical resistivity. Geotechnical Testing Journal.

[CR28] Kite G (1993). Computerized streamflow measurement using slug injection. Hydrological Processes.

[CR29] Kuyucak, N. (2002). Acid mine drainage prevention and control options. *CIM Bulletin*, 96–102.

[CR30] Li Y, Oldenburg DW (2000). 3-D inversion of induced polarization data. Geophysics.

[CR31] Li H, Shi A, Li M, Zhang X (2013). Effect of pH, temperature, dissolved oxygen, and flow rate of overlying water on heavy metals release from storm sewer sediments. Journal of Chemistry.

[CR32] Littlewood, I. G. (1987). Streamflow-pH dynamics in small moorland and conifer afforeste catchments in the upper Tywi valley, Wales. *National Hydrology Symposium,* British Hydrological Society*,* Hull, UK, 14–16 September 1987.

[CR33] Mao D, Revil A, Hort RD, Munakata-Marr J, Atekwana EA, Kulessa B (2015). Resistivity and self-potential tomography applied to groundwater remediation and contaminant plumes: sandbox and field experiments. Journal of Hydrology.

[CR34] Martínez-Pagán P, Cano ÁF, Aracil E, Arocena JM (2012). Electrical resistivity imaging revealed the spatial properties of mine tailing ponds in the Sierra Minera of Southeast Spain. Journal of Environmental & Engineering Geophysics.

[CR35] Martínez-Pagán P, Gómez-Ortiz D, Martín-Crespo T, Manteca JI, Rosique M (2013). The electrical resistivity tomography method in the detection of shallow mining cavities. A case study on the Victoria Cave, Cartagena (SE Spain). Engineering Geology.

[CR36] Matagi SV, Swai D, Mugabe R (1998). A review of heavy metal removal mechanisms in wetlands. African Journal of Tropical Hydrobiology and Fisheries.

[CR37] Mathias P (2001). The first industrial nation: the economic history of Britain 1700–1914.

[CR38] Mayes WM, Johnston D, Potter HAB, Jarvis AP (2009). A national strategy for identification, prioritisation and management of pollution from abandoned non-coal mine sites in England and Wales: methodology development and initial results. Science of the Total Environment.

[CR39] Mayes WM, Potter HAB, Jarvis AP (2010). Inventory of aquatic contaminant flux arising from historical metal mining in England and Wales. Science of the Total Environment.

[CR40] Michot D, Benderitter Y, Dorigny A, Nicoullaud B, King D, Tabbagh A (2003). Spatial and temporal monitoring of soil water content with an irrigated corn crop cover using surface electrical resistivity tomography. Water Resources Research.

[CR41] Mullinger N, Jarvis AP, Dudgeon BA, Younger PL (2004). Review of environmental and ecological impacts of drainage from abandoned mines in Wales. International Mine Water Association Symposium 1.

[CR42] Mungur AS, Shutes RBE, Revitt DM, House MA (1997). An assessment of metal removal by a laboratory scale wetland. Water Science and Technology.

[CR43] Navarro MC, Pérez-Sirvent C, Martínez-Sánchez MJ, Vidal J, Tovar PJ, Bech J (2008). Abandoned mine sites as a source of contamination by heavy metals: a case study in a semi-arid zone. Journal of Geochemical Exploration.

[CR44] Oldenburg DW, Li Y (1994). Inversion of induced polarization data. Geophysics.

[CR45] Parsons Brinkerhoff (2006) Remedial design for Esgair Mwyn Old Mine, Ceredigion, Wales: feasibility study*.* Parsons Brinkerhoff, Bristol, FSE96284A.

[CR46] Rey J, Martínez J, Hidalgo MC, Rojas D (2013). Heavy metal pollution in the Quaternary Garza basin: a multidisciplinary study of the environmental risks posed by mining, Linares, southern Spain. Catena.

[CR47] Reynolds, J. M. (2011). *An introduction to applied and environmental geophysics*. Wiley-Blackwell.

[CR48] Rhoades JD, Raats PAC, Prather RJ (1976). Effects of liquid-phase electrical conductivity, water content, and surface conductivity on bulk soil electrical conductivity. Soil Science Society of America Journal.

[CR49] Richardson JB (1974). Metal mining.

[CR50] Samouëlian A, Cousin I, Tabbagh A, Bruand A, Richard G (2005). Electrical resistivity survey in soil science: a review. Soil and Tillage Research.

[CR51] Schofield DI, Davies JR, Waters RA, Williams M, Wilson D (2009). A new Early Silurian turbidite system in Central Wales: insights into eustatic and tectonic controls on deposition in the southern Welsh Basin. Geological Magazine.

[CR52] Schwartz BF, Schreiber ME, Yan T (2008). Quantifying field-scale soil moisture using electrical resistivity imaging. Journal of Hydrology.

[CR53] Scorey, A. J. (2012). *Assessment of metal (Cd, Cu, Ni, Pb and Zn) contamination from an abandoned metal mine in Mid Wales, UK, to inform detailed remediation design.* MSc Thesis, Department of Geography, College of Science, Swansea University.

[CR54] Sheoran AS, Sheoran V (2006). Heavy metal removal mechanism of acid mine drainage in wetlands: a critical review. Minerals Engineering.

[CR55] Sidle RC, Chambers JC, Amacher MC (1991). Fate of heavy metals in an abandoned lead-zinc tailings pond: II Sediment. Journal of Environmental Quality.

[CR56] Skempton AW (1969). Consolidation of clays by gravitational compaction. Quaterly Journal of the Geological Society.

[CR57] SRK (1997) *Metal mines amelioration strategy: Esgair Mwyn.* SRK, Cardiff, PJKS/954PS001.

[CR58] Tabbagh A, Dabas M, Hesse A, Panissod C (2000). Soil resistivity: a non-invasive tool to map soil structure horizonation. Geoderma.

[CR59] Thompson S, Kulessa B, Luckman A (2012). Integrated electrical resistivity tomography (ERT) and self-potential (SP) techniques for assessing hydrological processes within glacial lake moraine dams. Journal of Glaciology.

[CR60] Thompson SS, Kulessa B, Benn DI, Mertes JR (2017). Anatomy of terminal moraine segments and implied lake stability on Ngozumpa Glacier, Nepal, from electrical resistivity tomography (ERT). Scientific Reports.

[CR61] Vereecken H, Binley A, Cassiani G, Revil A, Titov K (2006). Applied hydrogeophysics.

[CR62] Wardell Armstrong (1988) Preliminary report on the proposed tailings dump reprocessing operation at Esgair Mwyn Mine, Ffair Rhos, Tregaron, Dyfed*.* Wardell Armstrong, Cardiff, SAC/SDJ/W3107/1.

[CR63] Weller JM (1959). Compaction of sediments. AAPG Bulletin.

[CR64] Werth CJ, Zhang C, Brusseau ML, Oostrom M, Baumann T (2010). A review of non-invasive imaging methods and applications in contaminant hydrogeology research. Journal of Contaminant Hydrology.

[CR65] Younger PL (2000). The adoption and adaptation of passive treatment technologies for mine waters in the United Kingdom. Mine Water and the Environment.

[CR66] Zhou QY, Shimada J, Sato A (2001). Three-dimensional spatial and temporal monitoring of soil water content using electrical resistivity tomography. Water Resources Research.

